# Supplementary material for: What Types of Chemical Problems Benefit from Density-Corrected DFT? A Probe Using an Extensive and Chemically Diverse Test Suite
Source: J Chem Theory Comput. 2021 Feb 24;17(3):1368–79. doi: 10.1021/acs.jctc.0c01055 (PMC8028055; doi:10.1021/acs.jctc.0c01055)
Supplement: Supplementary file 1 — ct0c01055_si_001.pdf [file ct0c01055_si_001.pdf]

Electronic Supporting Information

What types of chemical problems benefit from density-corrected DFT? A probe using an extensive and chemically diverse test suite

*Golokesh Santra and Jan M.L. Martin**

Department of Organic Chemistry, Weizmann Institute of Science, 7610001 Reḥovot, Israel.

Email: gershom@weizmann.ac.il

Table S1: Abbreviations used and their descriptions

Abbreviation	Description
ACONF[1]	Relative energies of alkane conformers
ADIM6[2]	Interaction energies of n-alkane dimers
AHB21[3]	Interaction energies in anion–neutral dimers
AL2X6[4]	Dimerisation energies of AlX3 compounds
ALK8[4]	Dissociation and other reactions of alkaline compounds
ALKBDE10[5]	Dissociation energies in group-1 and -2 diatomics
AMINO20X4[6]	Relative energies in amino acid conformers
BH76RC[7]	Barrier heights of hydrogen transfer, heavy atom transfer, nucleophilic substitution, unimolecular and association reactions
BH76[8,9,7]	Reaction energies of the BH7610,11,23 set
BHDIV10[4]	Diverse reaction barrier heights
BHPERI[4,10,11,12]	Barrier heights of pericyclic reactions
BHROT27[4]	Barrier heights for rotation around single bonds
BSR36[13,14]	Bond-separation reactions of saturated hydrocarbons
BUT14DIOL[15]	Relative energies in butane-1,4-diol conformers
C60ISO[16]	Relative energies between C60 isomers
CARBHB12[4]	Hydrogen-bonded complexes between carbene analogues and H2O, NH3, or HCl
CDIE20[17]	Double-bond isomerisation energies in cyclic systems
CHB6[3]	Interaction energies in cation–neutral dimers
DARC[7,18]	Reaction energies of Diels–Alder reactions
DC13[19,7,20,21–29]	13 difficult cases for DFT methods
DIPCS10[4]	Double-ionisation potentials of closed-shell systems
FH51[30,31]	Reaction energies in various (in-)organic systems
G21EA[7,32]	Adiabatic electron affinities
G21IP[7,32]	Adiabatic ionisation potentials
G2RC[7,33]	Reaction energies of selected G2/97 systems
HAL59[34,35]	Binding energies in halogenated dimers (incl. halogen bonds)
HEAVY28[9]	Noncovalent interaction energies between heavy element hydrides
HEAVYSB11[4]	Dissociation energies in heavy-element compounds
ICONF[4]	Relative energies in conformers of inorganic systems
IDISP[7,36–39]	Intramolecular dispersion interactions
IL16[3]	Interaction energies in anion–cation dimers
INV24[40]	Inversion/racemisation barrier heights
ISO34[36]	Isomerisation energies of small and medium-sized organic molecules
ISOL24[41]	Isomerisation energies of large organic molecules
MB16-43[4]	Decomposition energies of artificial molecules
MCONF[42]	Relative energies in melatonin conformers
NBPRC[7,38,43]	Oligomerisations and H2 fragmentations of NH3/BH3 systems;H2 activation reactions with PH3/BH3 systems
PA26[4]	Adiabatic proton affinities (incl. of amino acids)
PArel[4]	Relative energies in protonated isomers
PCONF21	Relative energies in tri- and tetrapeptide conformers
PNICO23[44]	Interaction energies in pnicogen-containing dimers
PX13[45]	Proton-exchange barriers in H2O, NH3, and HF clusters
RC21[4]	Fragmentations and rearrangements in radical cations
RG18[4]	Interaction energies in rare-gas complexes

RSE43[46]	Radical-stabilisation energies
S22[47]	Binding energies of noncovalently bound dimers
S66[48]	Binding energies of noncovalently bound dimers
SCONF[7,49]	Relative energies of sugar conformers
SIE4X4[50]	Self-interaction-error related problems
TAUT15[4]	Relative energies in tautomers
UPU23[51]	Relative energies between RNA-backbone conformers
W4-11[52]	Total atomisation energies
WATER27[53]	Binding energies in (H2O)n, H+(H2O)n and OH−(H2O)n
WCPT18[54]	Proton-transfer barriers in uncatalysed and water-catalysed reactions
YBDE18[55]	Bond-dissociation energies in ylides

Table S2: Original and Optimized D4 parameters for HF-DFT and self-consistent DFT functionals together with five major subcategories of total WTMAD2

Functionals	s_6	a_1	s_8	a_2	Five top level subsets					WTMAD2
					THERMO[a]	BARRIERS[b]	LARGE[c]	CONF[d]	INTERMOL[e]	
HF-PBE-D4	1.0	0.5586	1.1145	3.6542	1.473	1.372	1.454	1.074	0.900	6.273
HF-PBE0-D4	1.0	0.4909	1.0402	4.2328	1.232	0.843	1.227	0.927	0.786	5.014
HF-PBE38-D4	1.0	0.4560	1.0815	4.5749	1.483	0.758	1.315	0.959	0.838	5.353
HF-PBE50-D4	1.0	0.4042	0.7455	4.7098	1.717	0.831	1.486	0.997	0.985	6.016
HF-BLYP-D4	1.0	0.4826	1.7143	3.2824	1.528	1.121	1.569	1.225	1.110	6.553
HF-B20LYP-D4	1.0	0.4215	1.2898	3.6437	1.351	0.663	1.222	0.994	0.869	5.100
HF-B3LYP-D4	1.0	0.4196	1.2556	3.7554	1.146	0.675	1.208	0.949	0.827	4.804
HF-B1LYP-D4	1.0	0.4102	1.1892	3.7353	1.279	0.600	1.188	0.939	0.835	4.840
HF-B38LYP-D4	1.0	0.3754	0.8643	3.8684	1.368	0.613	1.078	0.893	0.907	4.860
HF-BHLYP-D4	1.0	0.3624	0.6822	3.9886	1.563	0.879	1.069	0.988	1.000	5.499
HF-TPSS-D4	1.0	0.3303	1.3673	4.5177	1.424	0.891	1.566	0.853	0.975	5.709
HF-TPSSh-D4	1.0	0.5162	1.4287	3.7716	1.375	1.015	1.362	0.841	0.848	5.441
HF-TPSS0-D4	1.0	0.4743	1.2502	4.0521	1.449	0.817	1.214	0.755	0.859	5.094
HF-TPSS38-D4	1.0	0.4198	0.7804	4.1058	1.605	0.811	1.260	0.767	0.938	5.381
HF-TPSS50-D4	1.0	0.3928	0.7222	4.3802	1.894	0.932	1.387	0.885	1.027	6.125
HF-SCAN-D4	1.0	0.1586	0.7155	7.4456	1.303	1.045	1.075	0.900	0.757	5.079
HF-SCAN10-D4	1.0	0.2161	1.0159	7.3021	1.334	0.898	1.059	0.905	0.764	4.960
HF-SCAN0-D4	1.0	0.2290	1.0482	7.2144	1.509	0.786	1.141	0.949	0.787	5.172
HF-SCAN38-D4	1.0	0.2493	0.8788	6.9488	1.702	0.767	1.254	1.008	0.849	5.579
HF-SCAN50-D4	1.0	0.2810	0.5959	6.4527	1.947	0.849	1.374	1.067	0.943	6.181
HF-mPW1K-D4	1.0	1.1270	0.4163	4.4405	1.595	0.771	1.252	0.926	1.024	5.569
PBE-D4	1.0	0.7233	4.7411	4.7199	2.052	2.319	2.114	1.812	1.618	9.914
PBE-D4orig[1]	1.0	0.3857	0.9595	4.8069	2.078	2.407	1.946	1.879	2.117	10.426

[1] All “original” parameters are taken from ref[56,57].

PBE0-D4	1.0	0.5835	4.3194	5.3549	1.336	1.144	1.412	1.245	1.065	6.202
PBE0-D4orig[1]	1.0	0.4009	1.2007	5.0293	1.361	1.204	1.342	1.245	1.267	6.418
PBE38-D4	1.0	0.5208	2.8361	5.2438	1.482	0.762	1.331	1.098	0.981	5.653
PBE50-D4	1.0	0.4424	1.9249	5.3064	1.714	0.641	1.395	1.074	1.024	5.848
BLYP-D4	1.0	0.4702	3.6496	4.6263	2.062	1.931	2.461	1.629	1.282	9.365
BLYP-D4orig[1]	1.0	0.4449	2.3408	4.0933	2.006	2.066	2.188	1.851	1.493	9.603
B20LYP-D4	1.0	0.4355	2.1734	4.4258	1.4345	1.0656	1.7045	1.1361	0.9017	6.242
B3LYP-D4	1.0	0.4635	2.5224	4.5658	1.327	1.099	1.722	1.202	0.911	6.261
B3LYP-D4orig[1]	1.0	0.4087	2.0293	4.5381	1.301	1.124	1.610	1.235	1.096	6.366
B1LYP-D4	1.0	0.4296	1.8888	4.3733	1.376	0.900	1.548	1.045	0.849	5.717
B1LYP-D4orig[1]	1.0	0.3931	1.9855	4.5547	1.360	0.898	1.505	1.083	0.949	5.794
B38LYP-D4	1.0	0.3999	1.2738	4.2818	1.405	0.660	1.202	0.950	0.873	5.090
BHLYP-D4	1.0	0.3665	0.9499	4.4188	1.579	0.791	1.073	1.005	0.926	5.374
BHLYP-D4orig[1]	1.0	0.2726	1.6528	5.4863	1.601	0.839	1.192	1.074	0.940	5.646
TPSS-D4	1.0	0.5886	5.4374	5.0607	1.912	2.064	2.111	1.524	1.265	8.876
TPSS-D4orig[1]	1.0	0.4282	1.7660	4.5426	1.819	2.177	1.862	1.726	1.629	9.213
TPSSh-D4	1.0	0.5634	4.4400	4.9713	2.068	1.616	1.748	1.267	1.110	7.809
TPSSh-D4orig[1]	1.0	0.4429	1.8590	4.6023	1.577	1.694	1.593	1.391	1.307	7.562
TPSS0-D4	1.0	0.5050	3.3687	5.0625	1.496	1.045	1.340	0.992	0.969	5.842
TPSS0-D4orig[1]	1.0	0.4033	1.6244	4.8054	1.504	1.103	1.246	1.054	1.102	6.008
TPSS38-D4	1.0	0.4652	2.6661	5.1069	1.613	0.764	1.244	0.916	0.939	5.477
TPSS50-D4	1.0	0.4144	1.6981	5.0215	1.858	0.808	1.304	0.917	1.001	5.887
SCAN-D4	1.0	0.1898	3.0789	9.0102	2.330	1.895	1.360	1.226	1.594	8.404
SCAN-D4orig[1]	1.0	0.6293	1.4613	6.3128	2.331	1.898	1.347	1.237	1.708	8.521
SCAN10-D4	1.0	0.3149	6.4925	8.7697	1.531	1.510	1.228	1.105	1.419	6.793
SCAN0-D4	1.0	0.3750	6.1187	8.1124	1.549	1.040	1.192	1.001	1.198	5.980
SCAN38-D4	1.0	0.3996	5.0438	7.6249	1.691	0.768	1.256	0.997	1.075	5.788
SCAN50-D4	1.0	0.4108	3.2856	6.9783	1.901	0.754	1.377	1.055	1.051	6.138
mPW1K-D4	1.0	2.6336	0.4574	5.1437	1.506	0.603	1.220	1.042	1.038	5.409

[a]THERMO=Small Molecule Thermochemistry; [b]BARRIER=barrier heights; [c]LARGE=reaction energies for large systems; [d]CONF=conformer/intramolecular interactions; and [e]INTERMOL=intermolecular interactions

Table S3: Dispersion free HF-DFT and SC-DFT functionals together with five major subcategories of total WTMAD2

Functionals	Five top level subsets					WTMAD2
	THERMO	BARRIERS	LARGE	CONF	INTERMOL	
HF-PBE	1.570	1.132	2.316	4.601	4.222	13.840
HF-PBE0	1.246	0.698	1.821	4.048	3.885	11.697
HF-PBE38	1.458	0.706	1.730	3.802	3.704	11.400
HF-PBE50	1.671	0.875	1.786	3.575	3.580	11.487
HF-BLYP	2.212	0.907	3.429	6.676	8.807	22.030
HF-B20LYP	1.952	0.814	2.855	5.730	7.258	18.610
HF-B3LYP	1.660	0.784	2.719	5.485	6.800	17.446
HF-B1LYP	1.822	0.838	2.714	5.478	6.847	17.699
HF-B38LYP	1.784	1.023	2.426	4.956	5.942	16.131
HF-BHLYP	1.827	1.273	2.218	4.428	5.027	14.773
HF-TPSS	1.672	0.910	2.712	5.232	5.710	16.235
HF-TPSSh	1.555	0.750	2.440	4.941	5.410	15.095
HF-TPSS0	1.518	0.714	2.086	4.546	5.022	13.886
HF-TPSS38	1.608	0.849	1.933	4.241	4.713	13.345
HF-TPSS50	1.843	1.088	1.874	3.961	4.413	13.179
HF-SCAN	1.294	0.988	1.283	1.880	2.026	7.472
HF-SCAN10	1.331	0.846	1.252	1.930	2.019	7.377
HF-SCAN0	1.496	0.739	1.304	2.041	2.060	7.640
HF-SCAN38	1.683	0.738	1.403	2.153	2.116	8.092
HF-SCAN50	1.920	0.859	1.529	2.273	2.192	8.774
HF-mPW1K	1.577	0.819	1.777	4.286	4.845	13.304
PBE	2.083	2.194	2.640	3.817	3.157	13.891
PBE0	1.348	1.044	1.886	3.477	3.151	10.905
PBE38	1.466	0.692	1.720	3.389	3.217	10.484
PBE50	1.660	0.674	1.722	3.324	3.335	10.715
BLYP	2.373	1.904	3.732	5.707	7.141	20.857
B20LYP	1.777	1.129	2.949	5.120	6.215	17.190
B3LYP	1.586	1.151	2.823	4.810	5.635	16.005
B1LYP	1.709	0.989	2.766	4.938	5.927	16.328
B38LYP	1.694	0.857	2.386	4.584	5.304	14.824
BHLYP	1.776	1.036	2.112	4.193	4.634	13.751
TPSS	1.934	1.898	2.894	4.374	4.537	15.636
TPSSh	1.683	1.465	2.545	4.206	4.443	14.342
TPSS0	1.563	0.973	2.093	4.014	4.377	13.020
TPSS38	1.632	0.786	1.872	3.885	4.316	12.491
TPSS50	1.829	0.937	1.780	3.768	4.245	12.558
SCAN	2.308	1.865	1.481	1.576	2.169	9.400
SCAN10	1.522	1.476	1.357	1.598	2.098	8.051
SCAN0	1.540	0.995	1.322	1.668	2.020	7.545
SCAN38	1.672	0.734	1.391	1.815	2.048	7.660
SCAN50	1.875	0.762	1.516	2.030	2.134	8.316
mPW1K	1.497	0.617	1.722	3.951	4.447	12.234

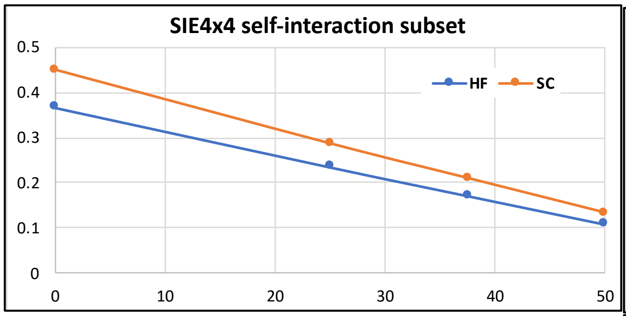
SIE4x4 self-interaction subset
0.5
0.4
0.3
0.2
0.1
0
0
10
20
30
40
50
HF
SC

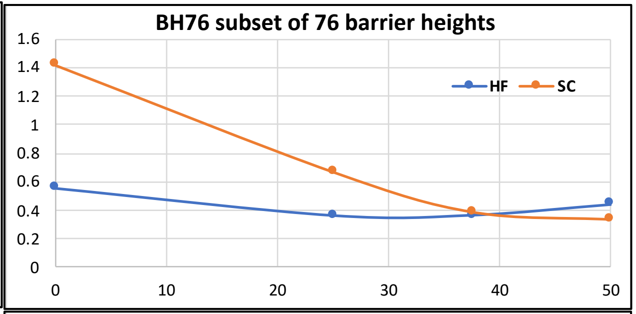
BH76 subset of 76 barrier heights
1.6
1.4
1.2
1
0.8
0.6
0.4
0.2
0
0
10
20
30
40
50
HF
SC

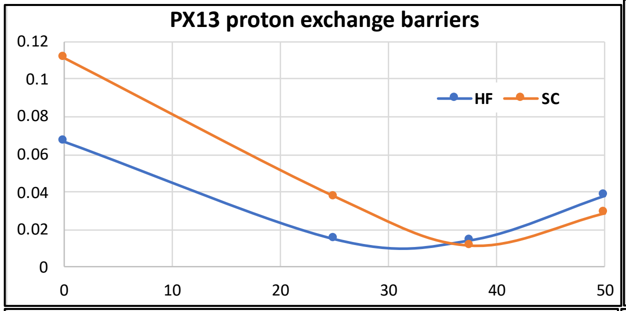
PX13 proton exchange barriers
0.12
0.1
0.08
0.06
0.04
0.02
0
0
10
20
30
40
50
HF
SC

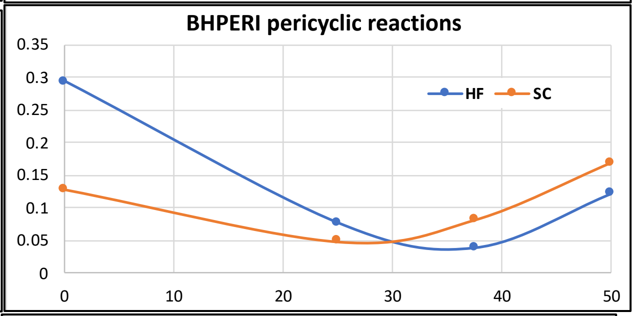
BHPERI pericyclic reactions
0.35
0.3
0.25
0.2
0.15
0.1
0.05
0
0
10
20
30
40
50
HF
SC

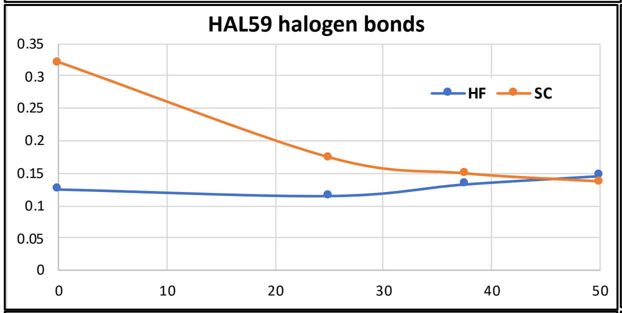
HAL59 halogen bonds
0.35
0.3
0.25
0.2
0.15
0.1
0.05
0
0
10
20
30
40
50
HF
SC

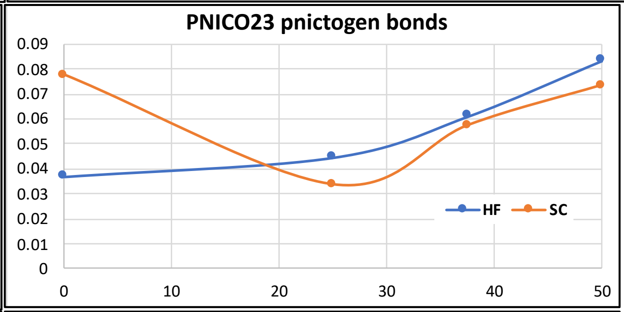
PNICO23 pnictogen bonds
0.09
0.08
0.07
0.06
0.05
0.04
0.03
0.02
0.01
0
0
10
20
30
40
50
HF
SC

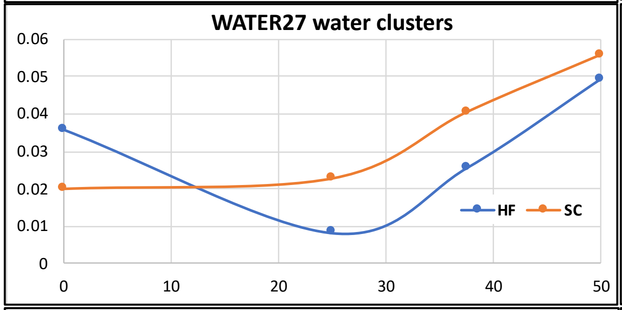
WATER27 water clusters
0.06
0.05
0.04
0.03
0.02
0.01
0
0
10
20
30
40
50
HF
SC

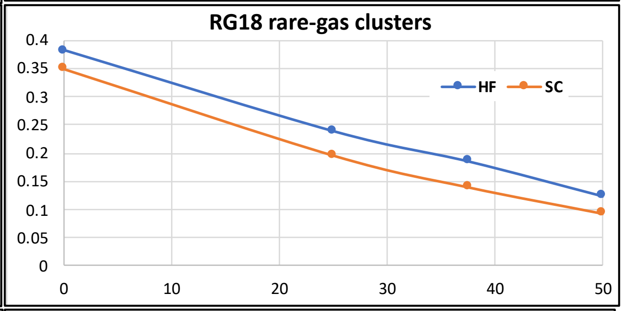
RG18 rare-gas clusters
0.4
0.35
0.3
0.25
0.2
0.15
0.1
0.05
0
0
10
20
30
40
50
HF
SC

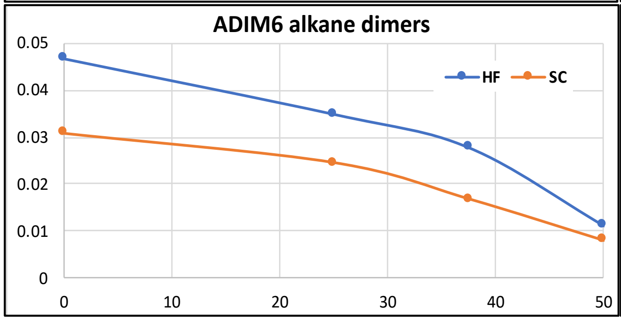
ADIM6 alkane dimers
0.05
0.04
0.03
0.02
0.01
0
0
10
20
30
40
50
HF
SC

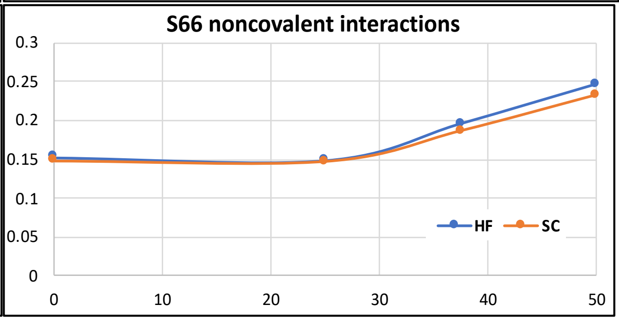
S66 noncovalent interactions
0.3
0.25
0.2
0.15
0.1
0.05
0
0
10
20
30
40
50
HF
SC

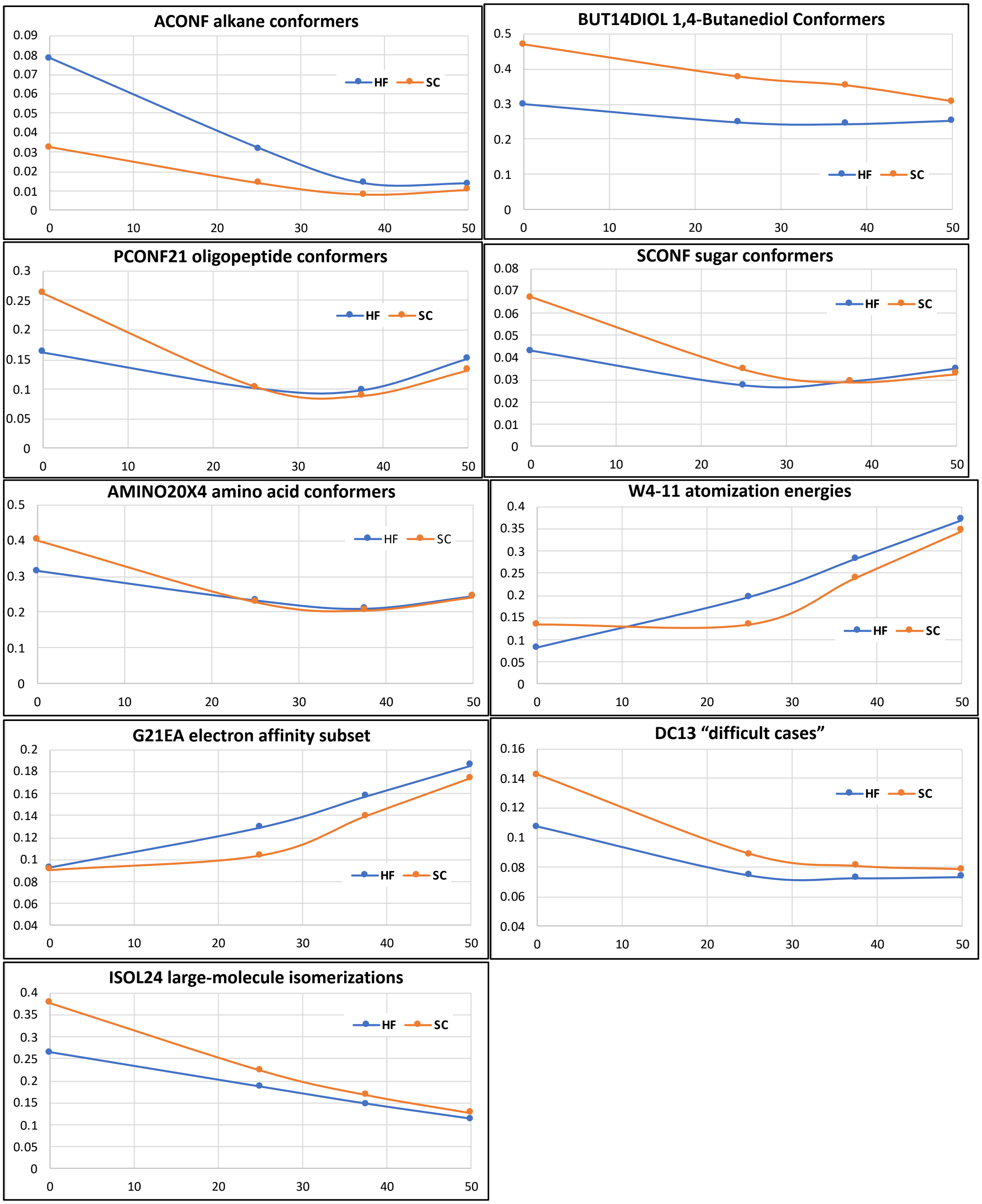

Figure S1: Dependence on the percentage of HF exchange, for self-consistent B*n*LYP-D4 and HF-B*n*LYP-D4 of the WTMAD2 (kcal/mol) contributions for the individual subsets SIE4x4, BH76, PX13, BHPERI, HAL59, PNICO23, W A TER27, RG18, ADIM6, S66, alkane conformers(ACONF), 1,4-butanediol conformers(BUT14DIOL), oligopeptide conformers

(PCONF21), sugar conformers(SCONF), amino acid conformers(AMINO20X4), G21EA, W4-11, DC13 and large molecule isomerization(ISOL24) subsets.

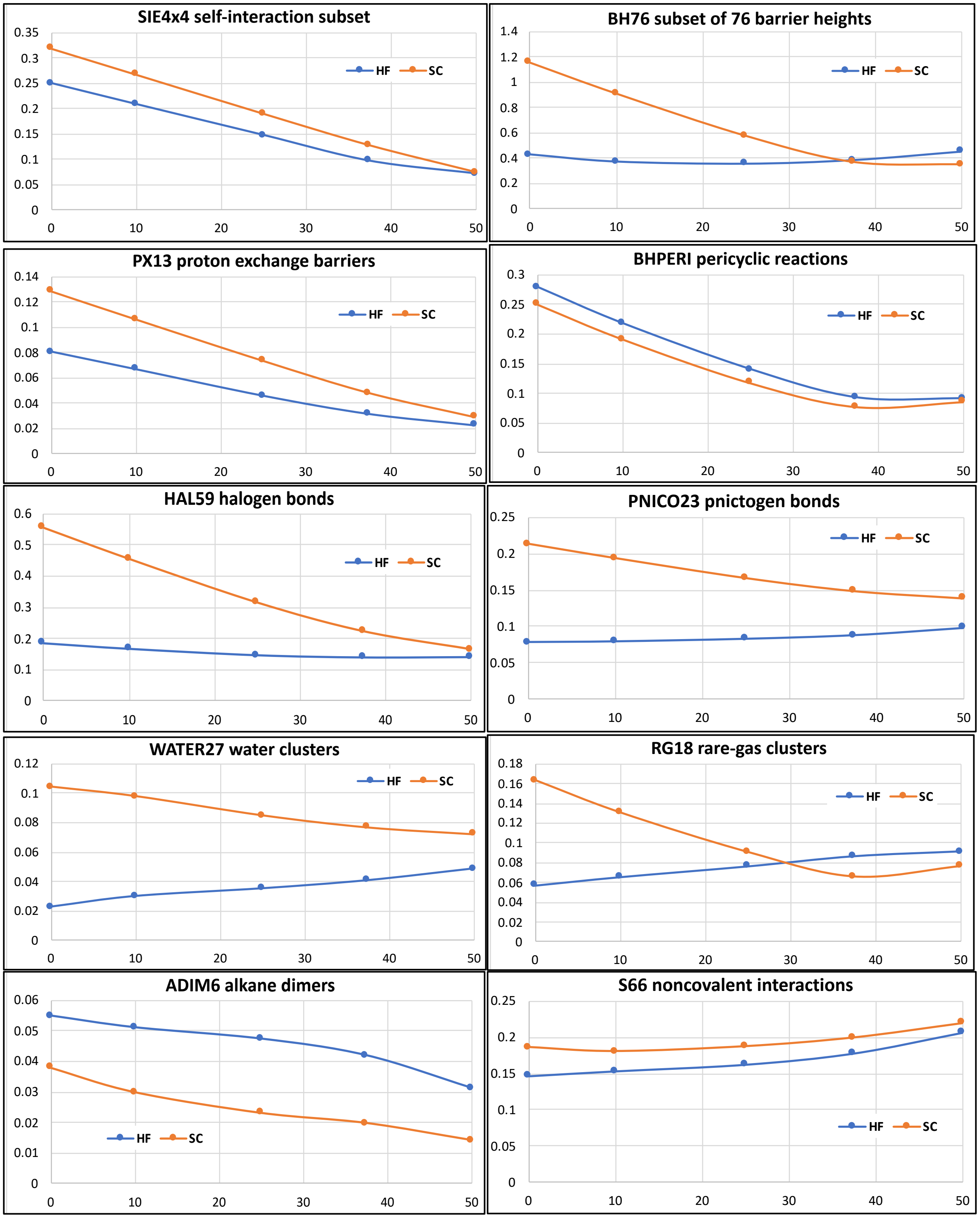

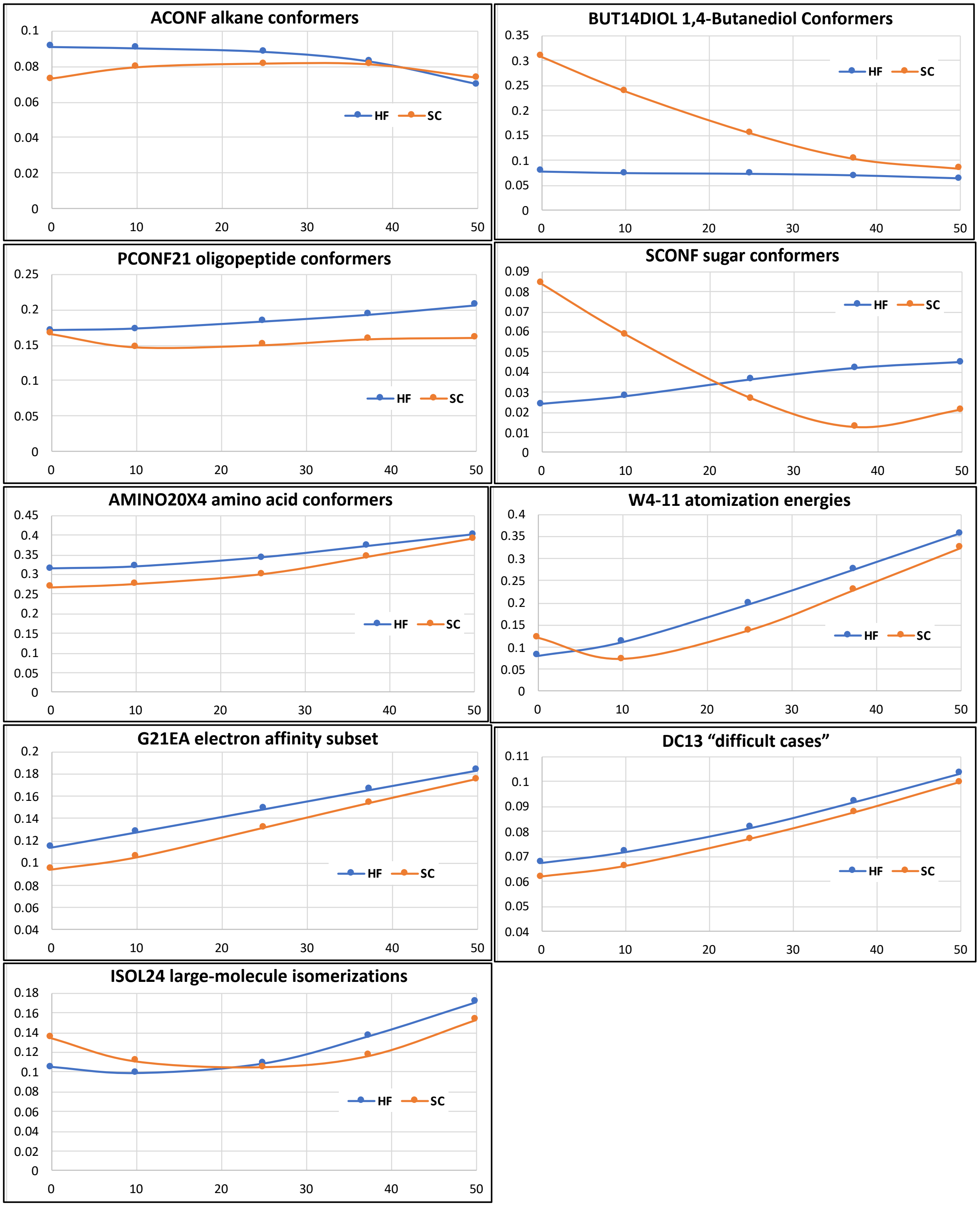

Figure S2: Dependence on the percentage of HF exchange, for self-consistent SCAN*n*-D4 and HF-SCAN*n*-D4 of the WTMAD2 (kcal/mol) contributions for the individual subsets SIE4x4, BH76, PX13, BHPERI, HAL59, PNICO23, W A TER27, RG18, ADIM6, S66, alkane conformers(ACONF), 1,4-butanediol conformers(BUT14DIOL), oligopeptide conformers (PCONF21), sugar conformers(SCONF), amino acid conformers(AMINO20X4), G21EA, W4-11, DC13 and large molecule isomerization(ISOL24) subsets.

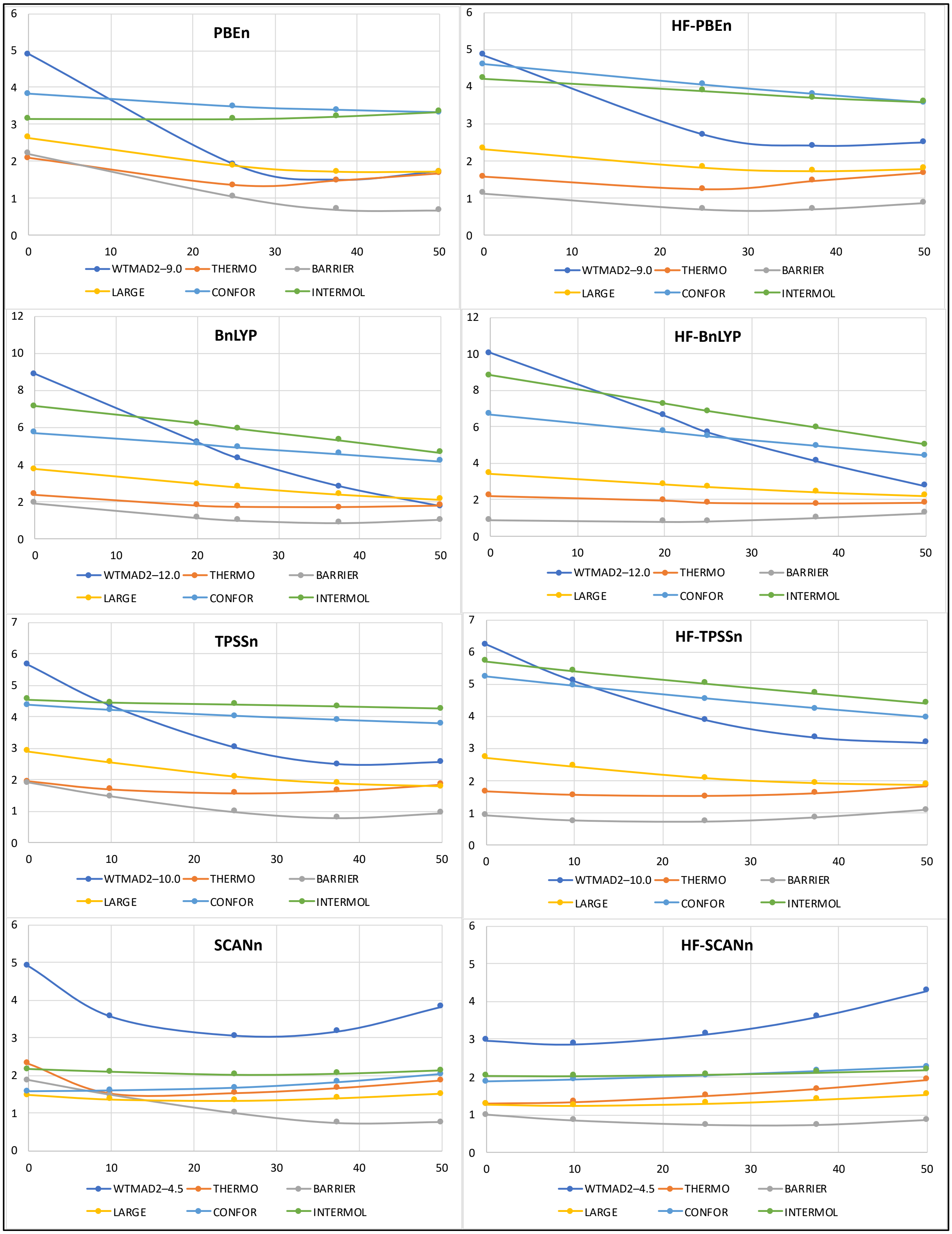

Figure S3: Dependence of WTMAD2 (kcal/mol) and of the five top-level subsets on the percentage of HF exchange(x-axis) for dispersion free PBE, BLYP, TPSS and SCAN series.

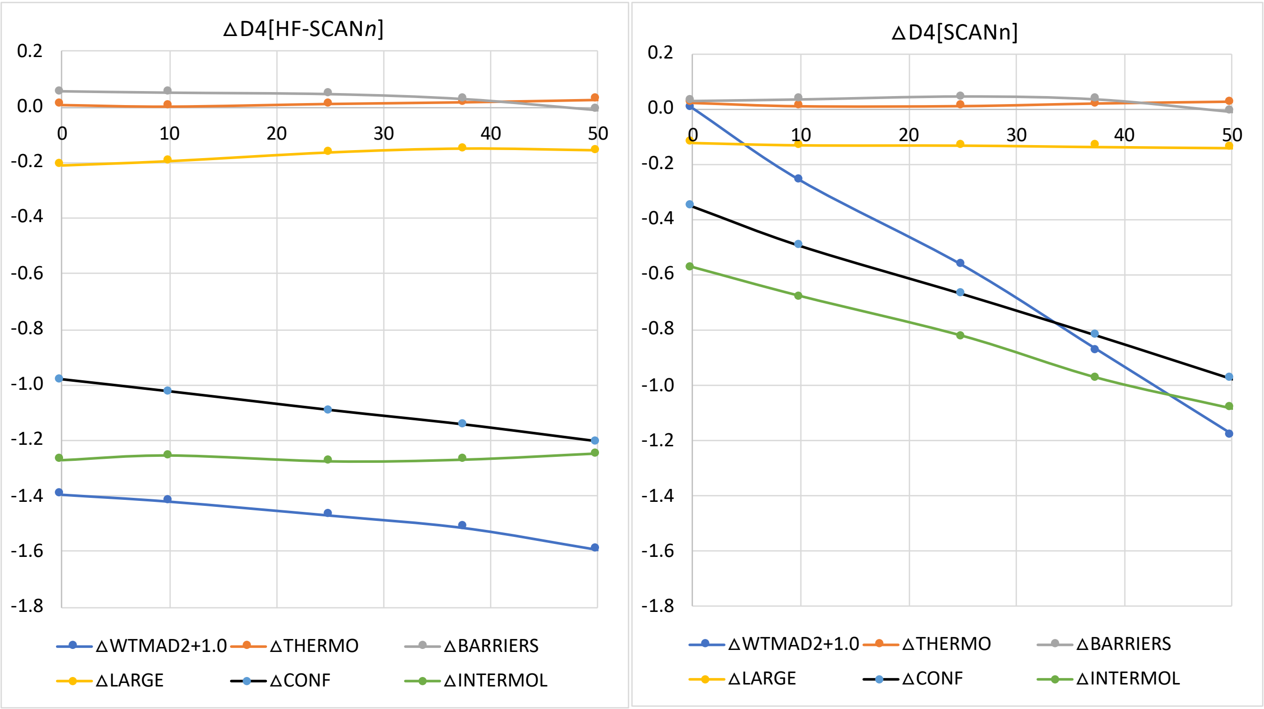

Figure S4: The effect of considering D4 dispersion correction on top of SCAN*n* and HF-SCAN*n* for different percentage of HF exchange.

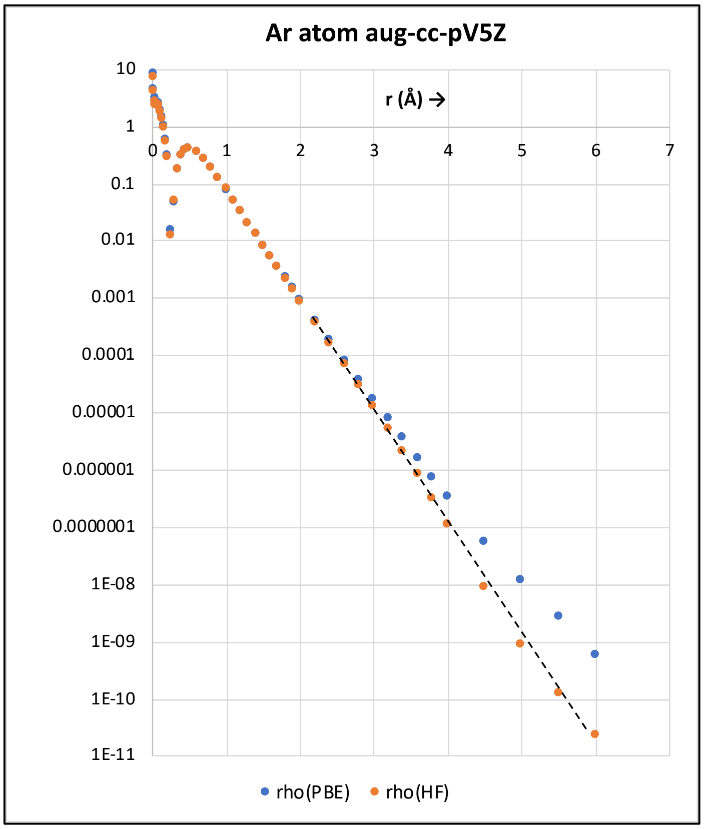

Figure S5: Decay trend of pure HF density and self-consistent PBE density for Ar atom

HF-DFT

A. Gaussian Sample Inputs:

1. PBE-D3BJ

```
%chk=file.chk
#p hf nosymm Def2QZVPP guess=save

title

0 1
 C    0.000000000000    0.000000000000     0.000000000000
 O    0.000000000000    0.000000000000     1.131400000000

--Link1—

%chk=file.chk
#p empiricaldispersion=gd3bj pbepbe Def2QZVPP scf(maxcycle=-1)
geom=allcheck guess=read
```

2. PBE38-D3BJ

```
%chk=file.chk
%mem=16gb
#p hf nosymm Def2QZVPP guess=save

title

0 1
 C    0.000000000000     0.000000000000     0.000000000000
 O    0.000000000000     0.000000000000     1.131400000000

--Link1—

%chk=file.chk
#p pbe1pbe iop(3/78=1000010000,3/76=0625003750,3/77=1000010000)
# empiricaldispersion=gd3bj
iop(3/174=1000000,3/175=1462300,3/177=0399500,3/178=5140500)
# iop(8/117=-100)
# scf(maxcycle=-1) chkbas geom=allcheck guess=read
```

B. ORCA Sample input:

1. TPSS-D3BJ

```
! UHF def2-QZVPP def2/J TightSCF NoPop XYZFILE

* xyz 0 1
 C    0.000000000000      0.000000000000     0.000000000000
 O    0.000000000000      0.000000000000     1.131400000000
*

$new_job
! UKS TPSS D3BJ def2-QZVPP def2/J grid5 moread NoPop

%scf
 maxiter 1
 tole 10000
 tolg 10000
end

* xyzfile 0 1
```

2. TPSS38

```
! UHF def2-QZVPP def2/JK TightSCF NoPop XYZFILE

* xyz 0 1
 C    0.000000000000    0.000000000000     0.000000000000
 O    0.000000000000    0.000000000000     1.131400000000
*

$new_job

! UKS TPSS0 def2-QZVPP def2/JK grid5 moread NoPop

%method
   Exchange X_TPSS
   Correlation C_TPSS
   ScalHFX 0.375
   ScalDFX 0.625
   ScalGGAC 1.0000
   ScalLDAC 1.0000
end
```

```
%scf
 maxiter 1
 tole 10000
 tolg 10000
end

* xyzfile 0 1
```

References:

(1) Gruzman, D.; Karton, A.; Martin, J. M. L. Performance of Ab Initio and Density Functional Methods for Conformational Equilibria of C n H 2 n +2 Alkane Isomers (n = 4−8) †. *J. Phys. Chem. A* **2009**, *113*, 11974–11983.

(2) Grimme, S.; Antony, J.; Ehrlich, S.; Krieg, H. A Consistent and Accurate Ab Initio Parametrization of Density Functional Dispersion Correction (DFT-D) for the 94 Elements H-Pu. *J. Chem. Phys.* **2010**, *132*, 154104.

(3) Lao, K. U.; Schäffer, R.; Jansen, G.; Herbert, J. M. Accurate Description of Intermolecular Interactions Involving Ions Using Symmetry-Adapted Perturbation Theory. *J. Chem. Theory Comput.* **2015**, *11*, 2473–2486.

(4) Goerigk, L.; Hansen, A.; Bauer, C.; Ehrlich, S.; Najibi, A.; Grimme, S. A Look at the Density Functional Theory Zoo with the Advanced GMTKN55 Database for General Main Group Thermochemistry, Kinetics and Noncovalent Interactions. *Phys. Chem. Chem. Phys.* **2017**, *19*, 32184–32215.

(5) Yu, H.; Truhlar, D. G. Components of the Bond Energy in Polar Diatomic Molecules, Radicals, and Ions Formed by Group-1 and Group-2 Metal Atoms. *J. Chem. Theory Comput.* **2015**, *11*, 2968–2983.

(6) Kesharwani, M. K.; Karton, A.; Martin, J. M. L. Benchmark Ab Initio Conformational Energies for the Proteinogenic Amino Acids through Explicitly Correlated Methods. Assessment of Density Functional Methods. *J. Chem. Theory Comput.* **2016**, *12*, 444–454.

(7) Goerigk, L.; Grimme, S. A General Database for Main Group Thermochemistry, Kinetics, and Noncovalent Interactions − Assessment of Common and Reparameterized (Meta -)GGA Density Functionals. *J. Chem. Theory Comput.* **2010**, *6*, 107–126.

(8) Zhao, Y.; Lynch, B. J.; Truhlar, D. G. Multi-Coefficient Extrapolated Density Functional Theory for Thermochemistry and Thermochemical Kinetics W. **2005**, 43–52.

(9) Zhao, Y.; González-García, N.; Truhlar, D. G. Benchmark Database of Barrier Heights for Heavy Atom Transfer, Nucleophilic Substitution, Association, and Unimolecular Reactions and Its Use to Test Theoretical Methods. *J. Phys. Chem. A* **2005**, *109*, 2012–2018.

(10) Guner, V.; Khuong, K. S.; Leach, A. G.; Lee, P. S.; Bartberger, M. D.; Houk, K. N. A Standard Set of Pericyclic Reactions of Hydrocarbons for the Benchmarking of Computational Methods: The Performance of Ab Initio, Density Functional, CASSCF, CASPT2, and CBS-QB3 Methods for the Prediction of Activation Barriers, Reaction

Energetics, And. *J. Phys. Chem. A* **2003**, *107*, 11445–11459.
(11) Ess, D. H.; Houk, K. N. Activation Energies of Pericyclic Reactions: Performance of DFT, MP2, and CBS-QB3 Methods for the Prediction of Activation Barriers and Reaction Energetics of 1,3-Dipolar Cycloadditions, and Revised Activation Enthalpies for a Standard Set of Hydrocarbon . *J. Phys. Chem. A* **2005**, *109*, 9542–9553.
(12) Dinadayalane, T. C.; Vijaya, R.; Smitha, A.; Sastry, G. N. Diels−Alder Reactivity of Butadiene and Cyclic Five-Membered Dienes ((CH) 4 X, X = CH 2 , SiH 2 , O, NH, PH, and S) with Ethylene: A Benchmark Study. *J. Phys. Chem. A* **2002**, *106*, 1627–1633.
(13) Steinmann, S. N.; Csonka, G.; Corminboeuf, C. Unified Inter- and Intramolecular Dispersion Correction Formula for Generalized Gradient Approximation Density Functional Theory. *J. Chem. Theory Comput.* **2009**, *5*, 2950–2958.
(14) Krieg, H.; Grimme, S. Thermochemical Benchmarking of Hydrocarbon Bond Separation Reaction Energies: Jacob's Ladder Is Not Reversed! *Mol. Phys.* **2010**, *108*, 2655–2666.
(15) Kozuch, S.; Bachrach, S. M.; Martin, J. M. L. Conformational Equilibria in Butane-1,4-Diol: A Benchmark of a Prototypical System with Strong Intramolecular H-Bonds. *J. Phys. Chem. A* **2014**, *118*, 293–303.
(16) Sure, R.; Hansen, A.; Schwerdtfeger, P.; Grimme, S. Comprehensive Theoretical Study of All 1812 C60isomers. *Phys. Chem. Chem. Phys.* **2017**, *19*, 14296–14305.
(17) Yu, L.-J.; Karton, A. Assessment of Theoretical Procedures for a Diverse Set of Isomerization Reactions Involving Double-Bond Migration in Conjugated Dienes. *Chem. Phys.* **2014**, *441*, 166–177.
(18) Johnson, E. R.; Mori-Sánchez, P.; Cohen, A. J.; Yang, W. Delocalization Errors in Density Functionals and Implications for Main-Group Thermochemistry. *J. Chem. Phys.* **2008**, *129*, 204112.
(19) Zhao, Y.; Truhlar, D. G. The M06 Suite of Density Functionals for Main Group Thermochemistry, Thermochemical Kinetics, Noncovalent Interactions, Excited States, and Transition Elements: Two New Functionals and Systematic Testing of Four M06-Class Functionals and 12 Other Function. *Theor. Chem. Acc.* **2008**, *120*, 215–241.
(20) Grimme, S. Semiempirical Hybrid Density Functional with Perturbative Second-Order Correlation. *J. Chem. Phys.* **2006**, *124*, 034108.
(21) Grimme, S.; Mück-Lichtenfeld, C.; Würthwein, E. U.; Ehlers, A. W.; Goumans, T. P. M.; Lammertsma, K. Consistent Theoretical Description of 1,3-Dipolar Cycloaddition Reactions. *J. Phys. Chem. A* **2006**, *110*, 2583–2586.
(22) Piacenza, M.; Grimme, S. Systematic Quantum Chemical Study of DNA-Base Tautomers. *J. Comput. Chem.* **2004**, *25*, 83–98.
(23) Woodcock, H. L.; Schaefer, H. F.; Schreiner, P. R. Problematic Energy Differences between Cumulenes and Poly-Ynes: Does This Point to a Systematic Improvement of Density Functional Theory? *J. Phys. Chem. A* **2002**, *106*, 11923–11931.
(24) Schreiner, P. R.; Fokin, A. A.; Pascal, R. A.; de Meijere, A. Many Density Functional Theory Approaches Fail To Give Reliable Large Hydrocarbon Isomer Energy Differences. *Org. Lett.* **2006**, *8*, 3635–3638.
(25) Lepetit, C.; Chermette, H.; Heully, J.; Lyon, D.; Uni, V.; Umr, C. Description of Carbo - Oxocarbons and Assessment of Exchange-Correlation Functionals for the DFT Description of Carbo -Mers. **2007**, 136–149.
(26) Lee, J. S. Accurate Ab Initio Binding Energies of Alkaline Earth Metal Clusters. *J. Phys. Chem. A* **2005**, *109*, 11927–11932.

(27) Karton, A.; Martin, J. M. L. Explicitly Correlated Benchmark Calculations on C 8 H 8 Isomer Energy Separations: How Accurate Are DFT, Double-Hybrid, and Composite Ab Initio Procedures? *Mol. Phys.* **2012**, *110*, 2477–2491.
(28) Zhao, Y.; Tishchenko, O.; Gour, J. R.; Li, W.; Lutz, J. J.; Piecuch, P.; Truhlar, D. G. Thermochemical Kinetics for Multireference Systems: Addition Reactions of Ozone. *J. Phys. Chem. A* **2009**, *113*, 5786–5799.
(29) Manna, D.; Martin, J. M. L. What Are the Ground State Structures of C 20 and C 24 ? An Explicitly Correlated Ab Initio Approach. **2016**.
(30) Friedrich, J.; Hänchen, J. Incremental CCSD(T)(F12*)|MP2: A Black Box Method To Obtain Highly Accurate Reaction Energies. *J. Chem. Theory Comput.* **2013**, *9*, 5381–5394.
(31) Friedrich, J. Efficient Calculation of Accurate Reaction Energies - Assessment of Different Models in Electronic Structure Theory. *J. Chem. Theory Comput.* **2015**, *11*, 3596–3609.
(32) Curtiss, L. A.; Raghavachari, K.; Trucks, G. W.; Pople, J. A. Gaussian-2 Theory for Molecular Energies of First- and Second-row Compounds. *J. Chem. Phys.* **1991**, *94*, 7221–7230.
(33) Curtiss, L. A.; Raghavachari, K.; Redfern, P. C.; Pople, J. A. Assessment of Gaussian-2 and Density Functional Theories for the Computation of Enthalpies of Formation. *J. Chem. Phys.* **1997**, *106*, 1063–1079.
(34) Kozuch, S.; Martin, J. M. L. Halogen Bonds: Benchmarks and Theoretical Analysis. *J. Chem. Theory Comput.* **2013**, *9*, 1918–1931.
(35) Řezáč, J.; Riley, K. E.; Hobza, P. Benchmark Calculations of Noncovalent Interactions of Halogenated Molecules. *J. Chem. Theory Comput.* **2012**, *8*, 4285–4292.
(36) Schwabe, T.; Grimme, S. Double-Hybrid Density Functionals with Long-Range Dispersion Corrections: Higher Accuracy and Extended Applicability. *Phys. Chem. Chem. Phys.* **2007**, *9*, 3397–3406.
(37) Grimme, S. Seemingly Simple Stereoelectronic Effects in Alkane Isomers and the Implications for Kohn–Sham Density Functional Theory. *Angew. Chemie Int. Ed.* **2006**, *45*, 4460–4464.
(38) Goerigk, L.; Grimme, S. Efficient and Accurate Double-Hybrid-Meta-GGA Density Functionals—Evaluation with the Extended GMTKN30 Database for General Main Group Thermochemistry, Kinetics, and Noncovalent Interactions. *J. Chem. Theory Comput.* **2011**, *7*, 291–309.
(39) Grimme, S.; Steinmetz, M.; Korth, M. How to Compute Isomerization Energies of Organic Molecules with Quantum Chemical Methods. *J. Org. Chem.* **2007**, *72*, 2118–2126.
(40) Goerigk, L.; Sharma, R. The INV24 Test Set: How Well Do Quantum-Chemical Methods Describe Inversion and Racemization Barriers? *Can. J. Chem.* **2016**, *94*, 1133–1143.
(41) Huenerbein, R.; Schirmer, B.; Moellmann, J.; Grimme, S. Effects of London Dispersion on the Isomerization Reactions of Large Organic Molecules: A Density Functional Benchmark Study. *Phys. Chem. Chem. Phys.* **2010**, *12*, 6940.
(42) Fogueri, U. R.; Kozuch, S.; Karton, A.; Martin, J. M. L. The Melatonin Conformer Space: Benchmark and Assessment of Wave Function and DFT Methods for a Paradigmatic Biological and Pharmacological Molecule. *J. Phys. Chem. A* **2013**, *117*, 2269–2277.
(43) Grimme, S.; Kruse, H.; Goerigk, L.; Erker, G. The Mechanism of Dihydrogen Activation

by Frustrated Lewis Pairs Revisited. *Angew. Chemie Int. Ed.* **2010**, *49*, 1402–1405.
(44) Setiawan, D.; Kraka, E.; Cremer, D. Strength of the Pnicogen Bond in Complexes Involving Group Va Elements N, P, and As. *J. Phys. Chem. A* **2015**, *119*, 1642–1656.
(45) Karton, A.; O'Reilly, R. J.; Chan, B.; Radom, L. Determination of Barrier Heights for Proton Exchange in Small Water, Ammonia, and Hydrogen Fluoride Clusters with G4(MP2)-Type, MPn, and SCS-MPn Procedures-a Caveat. *J. Chem. Theory Comput.* **2012**, *8*, 3128–3136.
(46) Neese, F.; Schwabe, T.; Kossmann, S.; Schirmer, B.; Grimme, S. Assessment of Orbital-Optimized, Spin-Component Scaled Second-Order Many-Body Perturbation Theory for Thermochemistry and Kinetics. *J. Chem. Theory Comput.* **2009**, *5*, 3060–3073.
(47) Jurečka, P.; Šponer, J.; Černý, J.; Hobza, P. Benchmark Database of Accurate (MP2 and CCSD(T) Complete Basis Set Limit) Interaction Energies of Small Model Complexes, DNA Base Pairs, and Amino Acid Pairs. *Phys. Chem. Chem. Phys.* **2006**, *8*, 1985–1993.
(48) Rezáč, J.; Riley, K. E.; Hobza, P. S66: A Well-Balanced Database of Benchmark Interaction Energies Relevant to Biomolecular Structures. *J. Chem. Theory Comput.* **2011**, *7*, 2427–2438.
(49) Csonka, G. I.; French, A. D.; Johnson, G. P.; Stortz, C. A. Evaluation of Density Functionals and Basis Sets for Carbohydrates. *J. Chem. Theory Comput.* **2009**, *5*, 679–692.
(50) Karton, A.; Rabinovich, E.; Martin, J. M. L.; Ruscic, B. W4 Theory for Computational Thermochemistry: In Pursuit of Confident Sub-KJ/Mol Predictions. *J. Chem. Phys.* **2006**, *125*, 144108.
(51) Kruse, H.; Mladek, A.; Gkionis, K.; Hansen, A.; Grimme, S.; Sponer, J. Quantum Chemical Benchmark Study on 46 RNA Backbone Families Using a Dinucleotide Unit. *J. Chem. Theory Comput.* **2015**, *11*, 4972–4991.
(52) Karton, A.; Daon, S.; Martin, J. M. L. W4-11: A High-Confidence Benchmark Dataset for Computational Thermochemistry Derived from First-Principles W4 Data. *Chem. Phys. Lett.* **2011**, *510*, 165–178.
(53) Bryantsev, V. S.; Diallo, M. S.; van Duin, A. C. T.; Goddard, W. A. Evaluation of B3LYP, X3LYP, and M06-Class Density Functionals for Predicting the Binding Energies of Neutral, Protonated, and Deprotonated Water Clusters. *J. Chem. Theory Comput.* **2009**, *5*, 1016–1026.
(54) Karton, A.; O'Reilly, R. J.; Radom, L. Assessment of Theoretical Procedures for Calculating Barrier Heights for a Diverse Set of Water-Catalyzed Proton-Transfer Reactions. *J. Phys. Chem. A* **2012**, *116*, 4211–4221.
(55) Zhao, Y.; Ng, H. T.; Peverati, R.; Truhlar, D. G. Benchmark Database for Ylidic Bond Dissociation Energies and Its Use for Assessments of Electronic Structure Methods. *J. Chem. Theory Comput.* **2012**, *8*, 2824–2834.
(56) Caldeweyher, E.; Ehlert, S.; Hansen, A.; Neugebauer, H.; Spicher, S.; Bannwarth, C.; Grimme, S. A Generally Applicable Atomic-Charge Dependent London Dispersion Correction. *J. Chem. Phys.* **2019**, *150*, 154122.
(57) Caldeweyher, E.; Bannwarth, C.; Grimme, S. Extension of the D3 Dispersion Coefficient Model. *J. Chem. Phys.* **2017**, *147*, 034112.
